# Recurrent Beta-Human Chorionic Gonadotropin (β-hCG) Elevation and Suspicion of Gestational Trophoblastic Neoplasia Following a Complete Hydatidiform Mole in a 17-Year-Old Female Patient: A Case Report

**DOI:** 10.7759/cureus.96387

**Published:** 2025-11-08

**Authors:** Amra Mujkanovic-Dzino, Bedrana Muracevic-Begovic, Rasim Iriskic

**Affiliations:** 1 Obstetrics and Gynecology, General Hospital Konjic, Konjic, BIH; 2 Obstretics and Gynecology, Cantonal Hospital Zenica, Zenica, BIH; 3 Obstetrics and Gynecology, Cantonal Hospital Zenica, Zenica, BIH

**Keywords:** adolescent, complete hydatidiform mole, epithelioid trophoblastic tumour, gestational trophoblastic neoplasia, molar pregnancy

## Abstract

The patients with complete hydatidiform mole (CHM) often present with markedly elevated beta-human chorionic gonadotropin (β-hCG) levels, uterine enlargement, and trophoblastic hyperplasia, and may develop complications such as preeclampsia, hyperthyroidism, or theca lutein cysts. The risk of developing persistent gestational trophoblastic neoplasia (GTN) following CHM is substantially higher than with partial hydatidiform mole (PHM). We describe the case of a 17-year-old female patient referred for evaluation of abdominal distension, abnormal vaginal bleeding, and hypertension. Imaging and laboratory findings suggested a molar pregnancy. She underwent repeat uterine evacuations and received methotrexate therapy. Histopathological examination confirmed a CHM. Serial monitoring initially demonstrated a decline in β-hCG, but subsequent fluctuations and recurrent vaginal bleeding raised suspicion for post-molar GTN. The additional histological findings raised the possibility of an epithelioid trophoblastic tumor. Following several cycles, β-hCG levels steadily declined and eventually normalized. This case highlights the diagnostic and therapeutic challenges of molar pregnancy in adolescents. It underscores the importance of β-hCG surveillance, timely initiation of chemotherapy when regression fails, and careful histopathological evaluation. Early recognition and individualized management can prevent complications and improve outcomes in young patients at risk of post-molar GTN.

## Introduction

Gestational trophoblastic disease (GTD) represents a spectrum of disorders arising from abnormal proliferation of trophoblastic tissue, ranging from benign hydatidiform moles to malignant gestational trophoblastic neoplasia (GTN) [1-3]. The hydatidiform mole is the most common manifestation, classified into complete hydatidiform mole (CHM) and partial hydatidiform mole (PHM) types according to genetic and histopathological characteristics [2-4].

A CHM is defined by the absence of embryonic or fetal tissue, diffuse villous swelling, and circumferential trophoblastic hyperplasia, whereas PHM often contains fetal elements and demonstrates focal trophoblastic proliferation [2,4,5]. Clinically, CHM often presents with abnormal vaginal bleeding, uterine size larger than expected for gestational age, markedly elevated beta-human chorionic gonadotropin (β-hCG) levels, and the presence of theca lutein ovarian cysts [3,6]. In cases of markedly elevated β-hCG, systemic complications may occur, including preeclampsia before 20 weeks, hyperthyroidism, and, in rare situations, acute respiratory distress syndrome [7,8].

Diagnosis integrates clinical presentation, laboratory findings, and imaging. Transvaginal ultrasound is usually the initial diagnostic modality, with a characteristic “snowstorm” or “cluster of grapes” pattern strongly indicative of CHM [9]. Serum β-hCG levels in molar pregnancies are often markedly higher than in normal pregnancies of comparable gestational age and are essential for both diagnosis and post-evacuation surveillance [3,10]. Histopathological examination of evacuated uterine tissue remains the gold standard for definitive confirmation [4,5].

The major concern following molar evacuation is progression to persistent GTN, which occurs in approximately 15%-20% of CHM cases and 1-5% of PHM cases [1,3,11]. Risk factors for malignant transformation include extremely elevated pre-evacuation β-hCG, large uterine size, advanced maternal age, and the presence of theca lutein cysts [6,7,12]. Rigorous follow-up with serial β-hCG monitoring is therefore critical. Rising or plateauing levels should prompt initiation of chemotherapy, most often with single-agent methotrexate or actinomycin D in low-risk disease [6,8,13].

We present the case of a 17-year-old girl diagnosed with CHM complicated by persistently elevated β-hCG. This case emphasizes the diagnostic and therapeutic challenges of molar pregnancy in adolescents and highlights the importance of close monitoring to prevent progression to GTN.

## Case presentation

A 17-year-old female patient was referred to the gynecology department by the pediatrician due to abnormal vaginal bleeding and a suspected abdominal mass. On initial examination, the patient was hypertensive (180/120 mmHg) and complained of headaches. She initially denied sexual activity and did not know the date of her last menstrual period. Laboratory tests revealed a decreased hemoglobin level and an elevated tumor marker cancer antigen 125 (CA-125; 55 U/mL). Consultations with internal medicine, neurology, and ophthalmology specialists were unremarkable.

Abdominal ultrasonography performed by a radiologist revealed a large, non-measurable intra-abdominal mass and a diffuse renal lesion with grade I hydronephrosis on the right side. The chest X-ray was normal. MRI of the abdomen and pelvis demonstrated a massive expansile lesion distending the uterine cavity, measuring 122x190x227 mm, consistent with a hypovascular neoplasm, along with bilateral grade II hydronephrosis and ureteral compromise at the pelvic entry.

**Figure 1:**
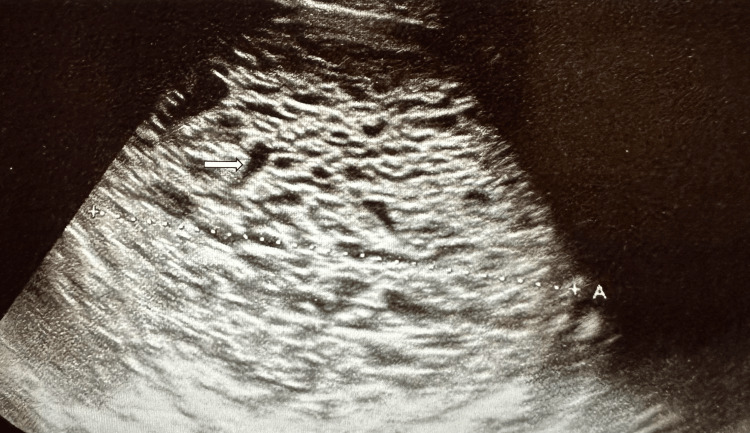
Transvaginal Ultrasound Showing a “Snowstorm” Appearance Ultrasound image of the uterus demonstrating the classic “snowstorm” pattern (white arrow), characteristic of a complete hydatidiform mole (CHM). The image shows diffusely enlarged, echogenic villous tissue without a viable fetus, consistent with the diagnosis of CHM.

On the sixth day of hospitalization, serum β-hCG was found to be markedly elevated (>150,000 mIU/mL), raising suspicion for molar pregnancy, which was subsequently confirmed by transvaginal ultrasonography (Figure 1). Upon disclosure of the results, the patient admitted to prior sexual activity. Due to profuse bleeding and significant anemia, she received three units of blood. The patient underwent uterine evacuation. A large volume of material was obtained and sent for histopathological examination, which confirmed a CHM. The endometrium thickness after the first curettage was 15 mm.

**Figure 2:**
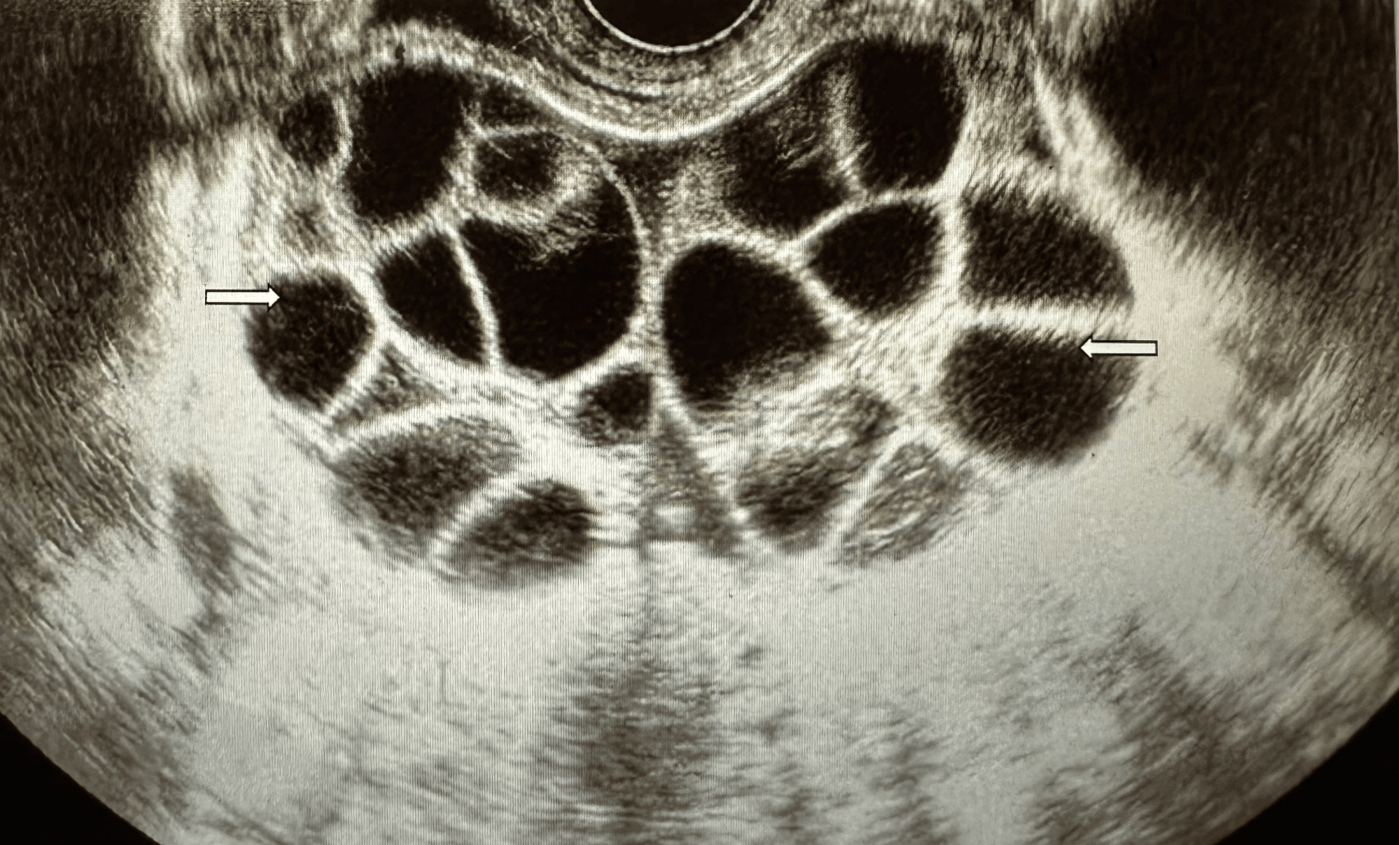
Ovarian Cysts Associated With Theca Lutein Hyper-Stimulation Ultrasound image showing bilateral ovarian enlargement with multiple cysts (white arrows), consistent with theca lutein cysts secondary to elevated β-hCG in molar pregnancy. The right ovary measured 108 × 73 mm, and the left ovary measured 84 × 75 mm. This finding reflects ovarian hyperstimulation associated with high β-hCG levels and is a recognized complication of complete hydatidiform mole.

Post-evacuation β-hCG levels were monitored closely: on day 20, β-hCG was 2,855 mIU/mL, but levels began to rise again (Table 1). Low-risk single-agent methotrexate therapy was initiated three weeks after the first evacuation. Due to persistently elevated β-hCG and unchanged ultrasonographic findings, including cystic ovarian enlargement (Figure 2), a repeat uterine curettage was performed. Histopathology findings after this intervention revealed inflamed decidual tissue with preserved focal trophoblastic cells and an absence of chorionic villi, without features of invasive disease. The findings were compatible with regressing post-molar changes. Serial chest X-rays remained unremarkable.

**Table 1:** Serial Beta-Human Chorionic Gonadotropin (β-hCG) Levels During Hospitalization (Days 6–55) Serial serum β-hCG levels during the initial hospitalization and treatment period. Values demonstrate the initial decline following uterine evacuation, transient increases, and response to methotrexate therapy and repeat curettage. The reference range for non-pregnant females is less than 5 mIU/mL. mIU/mL: milli-international units per milliliter

Day of Hospitalization	β-hCG (mIU/mL)	Reference Range (mIU/mL)
6	>150,000	<5 (non-pregnant)
8	72,996	<5
10	22,884	<5
12	7,532	<5
14	4,811	<5
16	3,192	<5
18	3,004	<5
20	2,855	<5
22	3,415	<5
24	4,177	<5
26	4,980	<5
28	4,776	<5
30	4,808	<5
32	2,958	<5
36	1,184	<5
38	721	<5
40	510	<5
45	261	<5
47	261	<5
49	229	<5
51	187	<5
53	79	<5
55	80	<5

The patient was discharged following the second curettage, with β-hCG levels stabilizing at 78 mIU/mL and the endometrium thickness at 10 mm. 

Two months later, the patient was readmitted with vaginal bleeding and lower abdominal pain. β-hCG had risen slightly (90 mIU/mL), and transvaginal ultrasonography showed cystically enlarged ovaries and an endometrial thickness of 13 mm. β-hCG continued to increase during hospitalization (90 → 118 → 144 mIU/mL), prompting another uterine curettage and evacuation of residual tissue (Table 2). Fifteen days post procedure, the β-hCG level was 170 mIU/mL. Oncology consultation raised suspicion for post-molar GTN. The final histopathology raised concern for an epithelioid trophoblastic tumor.

**Table 2:** Beta-Human Chorionic Gonadotropin (β-hCG) Levels During Follow-Up After Discharge and Readmission Serial serum β-hCG measurements following repeat uterine evacuation; a transient rise during readmission prompted oncology consultation, followed by normalization (<2.38 mIU/mL) at outpatient follow-up. Reference range for non-pregnant females: <5 mIU/mL. mIU/ml: milli- international units per milliliter

Follow-up Timepoint	β-hCG (mIU/mL)	Reference Range <5mIU/mL)	Notes
At discharge after repeat curettage	78	<5	Stable post-procedure
Readmission (2 months later)	90	<5	Mild increase, vaginal bleeding
Day 2 of readmission	118	<5	Rising trend
Day 3 of readmission	144	<5	Continuing rise
15 days post-second curettage	170	<5	β-hCG still increasing, oncology consulted
Discharge after oncology consult	30	<5	Outpatient follow-up recommended
First outpatient follow-up	<2.38	<5	Negative β-hCG, stable

The patient was discharged with a β-hCG of 30 mIU/mL and instructed to continue close outpatient oncological follow-up. On subsequent evaluation, β-hCG was <2.38 mIU/mL (negative), and hepatitis markers were negative. Given the patient’s clinical improvement, normalization of β-hCG, and lack of invasive features, a conservative surveillance approach was chosen rather than further surgical intervention. The patient continues to be monitored by oncology with serial β-hCG measurements every six to 12 months.

## Discussion

The CHM remains the most common form of GTD and carries a well-established risk of progression to GTN [1-3]. While the incidence of molar pregnancy has declined in high-income countries over the past decades, it continues to represent a significant clinical challenge in low- and middle-income regions due to delayed diagnosis and limited access to specialized care [4,5].

Adolescents and women over 40 years of age represent the groups at highest risk for molar pregnancy [3,6]. Our case highlights the unique challenges associated with managing GTD in an adolescent patient. Early recognition is critical because young patients often present with nonspecific symptoms, such as irregular bleeding or hyperemesis, which can delay diagnosis. Moreover, extremely elevated β-hCG levels, as observed in our case, are a well-documented predictor of post-molar GTN [2,7,8].

Ultrasound remains the first-line diagnostic tool, with the classic “snowstorm” appearance considered pathognomonic for CHM [9]. However, recent studies suggest that integrating advanced Doppler imaging and β-hCG kinetics may improve diagnostic accuracy, especially in atypical or partial forms [10,5]. Histopathological confirmation continues to be the gold standard [7]. In our case, histopathological examination following the final curettage demonstrated regressing trophoblastic activity and raised the possibility of an epithelioid trophoblastic tumor. However, the normalization of β-hCG and the patient’s stable clinical status supported conservative management and close surveillance rather than additional intervention.

The primary concern after uterine evacuation is the development of persistent GTN, which occurs in 15%-20% of CHM and up to 5% of PHM cases [1,3,11]. Predictors of malignant transformation include markedly elevated pre-evacuation β-hCG, excessive uterine size, and theca lutein cysts, all of which were present in our patient [6,8,12]. Current international guidelines recommend weekly β-hCG monitoring until three consecutive negative results, followed by monthly monitoring for at least six to 12 months [4,13].

Chemotherapy remains highly effective in GTN, with cure rates exceeding 90% even in high-risk disease. For low-risk patients, single-agent regimens with methotrexate or actinomycin D achieve remission in most cases [8,11]. In our case, methotrexate therapy was initiated promptly after plateauing β-hCG levels, consistent with standard practice. Early initiation of treatment is essential to prevent disease progression and minimize complications.

This case underscores the importance of careful follow-up and individualized management in young patients with molar pregnancy. Adolescents, in particular, may face psychosocial and reproductive challenges following GTD treatment. Fertility preservation remains a priority, and most patients are able to achieve successful pregnancies after treatment once surveillance is complete [5,12].

In summary, our case illustrates the diagnostic hallmarks and therapeutic considerations of CHM in an adolescent, emphasizing the importance of strict β-hCG surveillance, timely initiation of chemotherapy in cases of persistent disease, and the need for multidisciplinary support to optimize both oncological and reproductive outcomes.

## Conclusions

CHM in adolescents is a rare but clinically significant condition with a high risk of progression to GTN. Early recognition, strict surveillance with serial β-hCG monitoring, and timely initiation of chemotherapy are crucial for preventing complications and ensuring favorable outcomes. This case highlights the importance of multidisciplinary management and emphasizes that with appropriate treatment, fertility preservation and long-term prognosis remain excellent.
